# Visual and auditory reaction time for air traffic controllers using quantitative electroencephalograph (QEEG) data

**DOI:** 10.1007/s40708-014-0005-8

**Published:** 2014-10-10

**Authors:** Hussein A. Abbass, Jiangjun Tang, Mohamed Ellejmi, Stephen Kirby

**Affiliations:** 1 School of Engineering and Information Technology, University of New South Wales, Canberra, ACT 2600 Australia; 2 Department of Electrical and Computer Engineering, National University of Singapore, 4 Engineering Drive, Singapore, 117583 Singapore; 3 Eurocontrol Experimental Centre, Brétigny-sur-Orge, France

**Keywords:** Electroencephalography, Reaction time, Air traffic control

## Abstract

The use of quantitative electroencephalograph in the analysis of air traffic controllers' performance can reveal with a high temporal resolution those mental responses associated with different task demands. To understand the relationship between visual and auditory correct responses, reaction time, and the corresponding brain areas and functions, air traffic controllers were given an integrated visual and auditory continuous reaction task. Strong correlations were found between correct responses to the visual target and the theta band in the frontal lobe, the total power in the medial of the parietal lobe and the theta-to-beta ratio in the left side of the occipital lobe. Incorrect visual responses triggered activations in additional bands including the alpha band in the medial of the frontal and parietal lobes, and the Sensorimotor Rhythm in the medial of the parietal lobe. Controllers' responses to visual cues were found to be more accurate but slower than their corresponding performance on auditory cues. These results suggest that controllers are more susceptible to overload when more visual cues are used in the air traffic control system, and more errors are pruned as more auditory cues are used. Therefore, workload studies should be carried out to assess the usefulness of additional cues and their interactions with the air traffic control environment.

## Introduction

Quantitative electroencephalograph (QEEG) assessment of air traffic controllers (ATCOs) reveals objective insights into the mental processes and activities in response to task stimuli; and therefore, can lead to better understanding of the building blocks for their workload. The high temporal resolution of QEEG deems them suitable to monitor and assess performance objectively in real time.

Current air traffic control (ATC) graphical user interfaces use visual cues to communicate different information to the controller. For example, labels are used to communicate information on the individual aircraft, while change of an aircraft color or the color of a circle around an aircraft to a red color may indicate a potential violation of separation.

ATCOs rely significantly on these visual cues to assess the risk in the situation, while verbal communication is a common way to negotiate solutions and exchange information with pilots and other ATCOs. At times, the demand on visual and auditory processing can be high. For example, during conflict resolution, an ATCO would be communicating with a pilot to resolve a conflict while maintaining attention-focus on the scenario as it unfolds on the display. However, understanding the impact of auditory and visual cues on any ATCO’s mental processing is unclear.

Understanding this impact would help improve our understanding of visual and auditory performance on the one hand and workload, on the other hand. Hopkin [1] uses an example to demonstrate the interdependency, and sometimes conflict may arise between an ATCO’s workload and communication. In a light traffic situation, the ATCO may resort to increase the length of communication to fill up the time. While longer communication may get interpreted in the absence of context as an indication of high workload, only by isolating communication from workload, differences can be detectable. Understanding the contribution of visual and auditory activities to mental processes isolate their contributions from workload; thus providing a clearer picture of the main factors contributing to workload.

The performance [2] and sensorimotor association [3] of an individual can be measured by reaction time. This has the advantage that a reaction test is a non-invasive and non-intrusive test. When the measures are combined with QEEG analysis, the latter provides a means for validating the former, and it identifies brain functions associated with correct and incorrect reactions. These associations may provide the basis for a monitoring alarm system to act as a safety net against errors.

QEEG are non-intrusive. While they sense potentials from the scalp, they can reveal important high-order cognitive and behavioral information. Each EEG signal can be split into different bands for analysis, including Delta (1–4 Hz), Theta (4–8 Hz), Alpha (8–12 Hz), Beta (14–30 Hz), and Gamma (32–42 Hz). The power of an EEG signal indicates the number of neurons discharging synchronously; thus EEG power has the potential to reflect the capacity of cortical information processing [4]. However, measurements of EEG power are influenced with many elements including the thickness of the skull, volume of cerebrospinal fluid, technical issues including the type of montage, age, arousal, and cognitive demands during a task.

Different brain regions are known to be associated with one or more high-level cognitive tasks. For example, attention, memory, and executive control functions are normally seen in the prefrontal and frontal cortexes. The anterior of the medial is associated with flexibility, while the posterior of the medial is associated with memory making, orientation, and eye monitoring services. These are general observations gleaned from a wide range of papers. Translating these general observations into specific metric for brain activities is the subject of many studies in the literature including this paper.

Many studies focused on occipital activities. An increase in Beta activity is associated with a high degree of alertness [5, 6]. An increase in Alpha [7] is normally seen in relaxed individuals, while the power density of Alpha and Theta bands in $O_2$ and $P_4$ are appropriate indicators of sleepiness/wakefulness [8]. An increase in Theta activities in $O_1$ and $P_3$ may indicate slowness in information processing and a decrease in level of alertness [9–11].

Alpha (Theta) band power measured from different locations across the scalp is positively (negatively) correlated with cognitive performance and brain maturity [4]. That is, high alpha during rest is an indication of a healthy brain. During actual task demands, the Alpha band is suppressed more as cognitive and memory performances increase, while Theta shows the opposite behavior [4]. These are not universally accepted indicators [7, 12] and discrepancies exist among different studies. Therefore, there is an urge for more studies to better understand and map out the associations between different performance tasks and cognitive indicators.

One aspect that may contribute to discrepancy or disagreements among different studies is suggested by John & Easton [13]. They suggested that narrower bands less than 1 Hz are more suitable to measure workload. In theory, narrower bands increase the resolution of the signal in the frequency domain, where slower cycles between bands can be better identified. John & Easton [13] hypothesized that a very narrow band of EEG analysis can reveal more workload activities than a wide band. They conducted a series of experiments on the Visual and Auditory Continuous Pursuit Task (VACPT) and found that the visual unimodal pursuit activities were predominant in posterior temporal and parietal regions of the right hemisphere. The auditory unimodal pursuit activities were bilateral in anterior temporal and central regions. The multi-mode visual and auditory pursuit activities were predominant in all the four labeled frequencies in extensive frontal regions.

While John & Easton’s proposal of the usefulness of analyzing narrow bands is almost 19 years old, it did not gain much attention in the literature, probably because of the practical cost involved in doing the analysis. A question that is still to be answered is whether the qualitative differences [13] are significant enough to impact the results obtained from a quantitative analysis using a wide-band approach. Scerbo et.al. [14] raised this issue as a challenge for EEG studies in air traffic control because all studies have relied on a wide-band analysis.

The current paper presents the first investigation that compares narrow- and wide-band analysis on reaction tasks for air traffic controllers. The contribution of this study is twofold. First, previous studies on ATC used arbitrary wide bands. The current study is going to reveal if the use of wide bands can conceal information that are crucial for the analysis; thus, testing the hypothesis of John & Easton's [13]. Second, we present the first study that uses the Integrated Visual and Auditory (IVA) Continuous Performance Test (CPT) [15] for ATCOs.

This study is structured to understand mental load of air traffic controllers during a visual and auditory reaction test. Both narrow- and wide-band QEEG analyses are used to avoid a possible misinterpretation from using a wide-band analysis alone. A narrow-band analysis relies on a 1 Hz resolution or less. For example, if alpha is 8–12 Hz, a narrow-band analysis would create the following four levels of alpha bands: alpha-very-low (8 to $< 9$ Hz), alpha-low (9 to $< 10$ Hz), alpha-high (10 to $< 11$ Hz), and alpha-very-high (11 to $< 12$ Hz).

## Methods

### The task

A variation of the IVA-CPT [15] was used. The original IVA (www.braintrain.com) is a 13-min attention test and is normally used to assess subjects with attention deficit hyperactivity disorders before and after neurofeedback training sessions.

Subjects are instructed to press the left-button of the mouse when they hear or see a “1” and that they should not press any button when they hear or see a “2”. Subjects were told that the appearance or uttering of either numbers are random; therefore, the same number could get displayed or spoken a number of times in a sequence. In case of visual stimuli, the same number may disappear and reappear again consecutively.

**Fig. 1:**
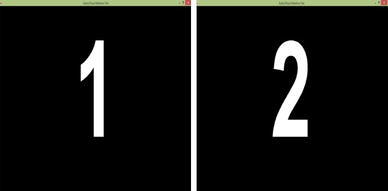
The visual cues used during the experiment

The visual stimuli (1 or 2) were displayed in the middle of the screen using a large white color on a black background and 389pts font size as shown in Fig. 1. The auditory stimuli (1 or 2) were presented using an external speaker with a machine voice, the volume was maintained constant during all experiments at a sound level of approximately 50 dB measured at 1 m from the speakers.

Each stimulus (visual or auditory) lasted between 500 and 600 ms, followed by a pause for 10 ms. To redraw a visual stimuli, the previous image needs to be cleared. The time taken to clear the previous image and draw a new one took 1.89 ms on average. To utter the auditory signal, the voice was cashed at the time the program started and it took on average 1.03 ms to retrieve the cashed auditory signal every time it is used.

Each participant was given a demonstration of the task. Each session lasted for 6 min and was divided into twelve half-minute blocks. Ten blocks excluding the first (warm-up block) and last (cool-down block) are used for analysis. Each block contained 50–60 trials. Trials were continuous during each session with no breakout periods in between the trials.

IVA-CPT normally balances auditory and visual targets. In this work, the test was modified by varying the distribution for the auditory and visual targets between the blocks. Therefore, while the classic test uses the same distribution for auditory and visual targets within a single block, we used different distributions to induce different loads during the test.

With p1/p2 representing the probability to see or hear “1”, respectively, the distribution of visual and auditory targets in each of the ten blocks was: 0.5/0.5, 0.8/0.8, 0.5/0.5, 0.2/0.2, 0.5/0.5, 0.2/0.8, 0.5/0.5, 0.8/0.2, 0.5/0.5, and 0.8/0.8. These blocks were presented sequentially to be able to measure differences in loads of particular stimuli by subtracting the differences in performance of the 50–50 loads. For example, starting with 50 % visual target and 50 % auditory target, switching to 80–80, then switching back to 50–50, we can contribute the difference between the first 50–50 block and the third 50–50 block to the load of the second 80–80 block.

### Participants

Obtaining subjects in ATC is a very expensive exercise because of the smaller population and the cost involved in recruiting highly specialized controllers. Therefore, it is common to conduct studies with a couple of controllers. In this study, we were fortunate to have five very experienced male controllers (Avg age 52.5) with average experience of 18 years. They all used their right-hand in controlling the mouse, had normal or corrected-to-normal vision, and were free of known hearing impairments. All participants gave a written informed consent to participate in this study. The data used for the analysis in this paper is a subset of a much larger experiment (see [16, 17] for more details).

Participants were seated in front of a Core i7, 2GHz Windows 8, 64-bit, 8GB RAM laptop. The display was placed at eye level with an approximate viewing distance of 65cm. Responses were recorded from pressing the left button of a two-button ergonomic mouse.

### Data acquisition and analysis system

The $\text {Nexus}32^{TM}$ QEEG system and a cap were used, with 19 electrodes, two references (an electrode under each earlobe close to retrahens Auricular), and 1 ground (an electrode positioned at AFz) following the 10–20 standard. The sampling rate was 2,048 Hz. The high sampling rate was used to allow flexibility when analyzing event-related potentials. Subjects were prepared by first cleaning the electrode positions on the scalp using alcohol pads. The cap was then mounted, adjusted, and the electrodes were filled with appropriate amount of skin prepping gel. After ensuring that all signals are of high quality (electrode offset is between $\pm 50,000$), the session starts. During the session, a screen indicating the quality of the signal for each electrode was used to monitor signal quality during data collection. A green color was used to indicate high quality signals, while a red color was used as an alarm for possible problems. No alarm was flagged during any of the experiments reported in this paper. EEG analysis was done on a second-by-second basis then averaged over 30 s for each IVA-CPT block.

The IVA-CPT responses were time-tagged and were saved on the computer. The same machine was used for EEG data collection to ensure appropriate clock synchronization between the EEG data and responses from IVA. All EEG signals were band-pass filtered between 1 and 42 Hz. Artifacts were visually detected from the phenotype of the signal.

Matlab 2014 and Excel 2007 were used for the processing of the data and the generation of table summaries, including statistical analysis, respectively. The data acquisition and real-time analysis software was written in C3 and the EEGlab Matlab code (http://sccn.ucsd.edu/eeglab/) was used for visualization.

## Results and discussion

### Performance and reaction time

Response time is recorded for both target and non-target (Table 1). A response to a non-target is when a subject presses the left button of the mouse when he should not have. This happens in the case of the number “2” appearing or gets spoken and the subject could not inhibit his reaction; instead he presses the left button of the mouse.

**Table 1:** Average and standard-deviation of the response time on visual and auditory stimuli

	S1	S2	S3	S4	S5
Visual 1	344 $\pm$ 192	373 $\pm$ 194	370 $\pm$ 177	364 $\pm$ 165	268 $\pm$ 176
Auditory 1	280 $\pm$ 190	178 $\pm$ 181	206 $\pm$ 139	271 $\pm$ 197	213 $\pm$ 134
Visual 2	182 $\pm$ 107	168 $\pm$ 156	169 $\pm$ 119	171 $\pm$ 135	176 $\pm$ 156
Auditory 2	189 $\pm$ 137	152 $\pm$ 149	204 $\pm$ 122	163 $\pm$ 159	205 $\pm$ 142

Previous studies have shown that the time of accurate response to visual targets is slower than the time of accurate response to auditory targets [18]. The reaction times to auditory targets are consistent with previous studies [19] but the results of visual targets were 1.5 more than Niruba & Maruthy [19] results. However, both results are consistent with Shelton & Kumar [20] where they found that the reaction time on simple visual stimuli was 331 ms, while the reaction time to auditory stimuli was 284 on a control group of healthy people.

The reaction time when a controller makes a mistake for both visual and auditory stimuli is of similar values. There is no significant difference between the visual and auditory cases using a one-tail student's t-test $(\rho = 0.05)$. The reaction time when making a mistake is always faster than when not making a mistake. However, because of the variance in reaction time, the test of significance results is inconclusive.

Performance is measured by accuracy on target (correct responses to visual/auditory targets), false positive (false alarm or failure to inhibit a motor task, wrong mouse clicks), and false negatives (misses or incorrect inhibition of a motor task, mouse click).

**Table 2:** Pearson correlation between reaction time and performance

Performance		Reaction time	
		Visual target	Auditory target
Accuracy on target			−0.32
Visual Auditory		0.48 0.27	
False positive			0.22
Visual Auditory		−0.38 −0.25	
False negative			−0.45
Visual Auditory		−0.22 0.22	

Some strong correlations were found between reaction time and performance as measured by subjects’ correct/incorrect responses to targets (Table 2). The summary of these results is:

- Slow visual reaction time is associated with an increase in correct visual response, and a decrease in unintended visual response and visual response inhibition.
- Slow visual reaction time is associated with an increase in correct auditory response and in auditory response inhibition, and a decrease in unintended auditory response.
- Slow auditory reaction time is associated with a decrease in correct auditory response and auditory response inhibition, and an increase in unintended auditory response.

The results related to visual responses can probably be explained by the expected delays associated with a visual stimulus as a result of the existence of many collateral pathways to various associated areas [19].

### Performance and EEG bands

The correlations between all EEG channels and their bands (133 vectors), and the three performance measures were calculated. Significant high correlations exceeding 0.5 and $(\rho < 0.001)$ are extracted and shown in Table 3. No high correlations were found between the performance on auditory tasks and the EEG channels and bands.

**Table 3:** Correlation between performance and EEG Channels/bands

Channel	Band	Accuracy on target	Wrong inhibition of a motor task
F3	Theta	0.60	0.69
Fz	Theta	0.61	0.69
F4	Theta	0.62	0.68
O1	Theta-to-Beta	0.62	0.68
Pz	Total Power	0.64	0.7
Fz	Alpha		0.61
Pz	Alpha		0.66
Pz	SMR		0.65

The channels and bands with significant high correlations when accurate identification of visual targets occurred are also activated when wrong inhibition of a motor task occurred. However, extra areas with significant high correlation appear when the user makes a mistake; mainly associated with the Alpha and SMR bands.

Theta power in the frontal lobe, Theta-to-Beta ratio in the left occipital lobe, and total power at the medial of the parietal lobe were positively correlated to correct reaction to the visual target. Additional positive correlations with the alpha band in the medial of the frontal and parietal lobes as well as SMR in the medial of the parietal lobe emerged when the subjects inhibited response by mistake.

Klimesch [4] showed that Alpha gets suppressed as cognitive and memory performance increased, while Theta showed the opposite behavior. The results support Klimesch's results and extend them by demonstrating that an increase in alpha occurs with incorrect inhibition of a response (when the subject does not press the button when he should have). These alpha bursts when a mistake occurs require more detailed analysis.

### Narrow- or wide-band analysis

**Fig. 2:**
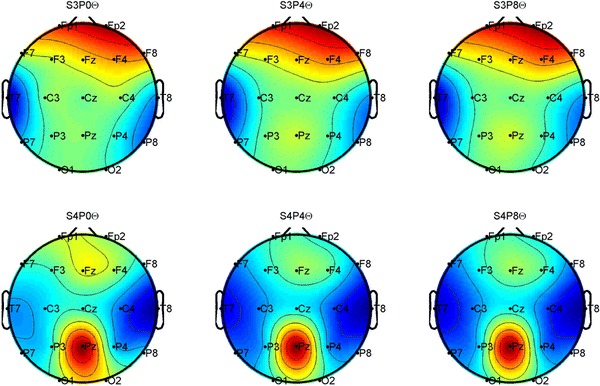
A topographical map of Theta power for one subject (*top*) and another subject (*bottom*). Band resolutions were 1 Hz (on *left*), 0.25 Hz (*middle*), and 0.125 Hz (*right*)

Qualitative differences exist when visualizing the distribution of power using different resolutions for spectrum analysis. For example, there is a difference in the power in the medial of frontal lobe for the subject visualized in the bottom of the diagram (Fig. 2) when a resolution of 1 Hz is used (most left plot) and lower resolutions were used (middle and right plot). Similarly, small qualitative differences exist in Pz for the subject visualized in the top row.

There were no qualitative differences between blocks. The same distribution of power in each band was qualitatively identical across blocks for the same resolution used for spectrum analysis.

While slight qualitative differences existed between a resolution of 1 and the other two finer resolutions of 0.25 and 0.125, the correlation between performance and EEG presented in the previous sub-section was identical in all three resolutions.

This result confirms John & Easton's [13] work that there are qualitative differences that can be seen in the visualization, but within the scope of this experiment, there were no advantages in using narrow bands over wide bands.

## Conclusion

The reaction time and performance of air traffic controllers were assessed using the IVA-CPT. Nineteen EEG channels using the 10–20 standard were used to monitor EEG during the task.

Three main findings of this study can be summarized as follows:

- Reaction time to visual targets was slower than to auditory ones and was positively correlated to performance on both visual and auditory targets.
- The same brain areas that get activated in the events associated with correct responses to visual targets also get activated when incorrect responses occur. However, three additional areas are activated in the latter case. We hypothesize that alpha wave activities in the medial—more specifically $F_z$ and $P_z$—can potentially be used to infer a wrong response.
- While qualitative differences may exist when analyzing the EEG data using narrow bands, no quantitative advantage was seen in our analysis. Therefore, the claim that narrow-band analysis will provide different results is questionable.

The conclusion of this study for air traffic control is twofold. First, overloading air traffic controllers with visual cues can cause an increase in their workload because of the more complex pathways used in visual processing causing slower response to visual targets. Second, QEEG has the potential to be used as an auxiliary mechanism to monitor workload, and possibly detect incorrect reactions to stimuli during an ATC task.

For future work, we will calibrate the results obtained from this study, where reaction time was analyzed, with the wider massive dataset we collected during the experiment. Further analysis will be conducted to gain insight into the performance of air traffic controllers through objective QEEG data.
